# Factors associated with wasting and stunting among children aged 06–59 months in South Ari District, Southern Ethiopia: a community-based cross-sectional study

**DOI:** 10.1186/s40795-023-00683-3

**Published:** 2023-02-24

**Authors:** Temesgen Mohammed Toma, Kassahun Tamene Andargie, Rahel Abera Alula, Bahiru Mulatu Kebede, Mintesinot Melka Gujo

**Affiliations:** 1 Department of Public Health, Arba Minch College of Health Sciences, P.O.Box: 155, Arba Minch, Ethiopia; 2 Department of Nursing, Arba Minch College of Health Sciences, P.O.Box: 155, Arba Minch, Ethiopia; 3 Southern Region Health Bureau Public Health Institute, Hawassa, Ethiopia

**Keywords:** Wasting, Stunting, Factors, Children aged 06–59 months, Ethiopia

## Abstract

**Background:**

Child undernutrition is a major public health problem in Ethiopia despite different nutrition-specific and sensitive interventions implemented by the government. However, evidence regarding the burden and responsible factors is limited in the South Ari district. Hence, this study aimed to assess factors associated with wasting and stunting among children aged 06–59 months in the South Ari district, Southern Ethiopia, 2021.

**Methods:**

A community-based cross-sectional study was conducted from March 11 to April 11, 2021, among 717 households with at least one child aged 06–59 months. Anthropometric measurements were taken using standard procedures and were converted to height for age Z score and weight-for-height using WHO Anthro software Version 3.2.2. Data were checked and entered into Epi-Data Version 3.1 and analyzed using SPSS Version 25.0. Binary logistic regression analysis was fitted to identify predictors of wasting and stunting. A *p*-value < 0.05 was used to declare statistical significance.

**Result:**

The prevalence of wasting and stunting among children aged 06–59 months in the district was 9.1% (95% CI: 7.07%, 11.41%) and 59.97% (95% CI: 56.28%, 63.58%), respectively. Family size (8 and above) (AOR = 3.03, 95% CI: 1.31, 7.03), family size (5 to 7) (AOR = 2.05, 95% CI: 1.11, 3.81), poor and medium wealth index (AOR = 3.69, 95% CI: 1.65, 8.26) and (AOR = 2.29, 95% CI: 1.01, 5.16), insufficient maternal knowledge on child feeding practice (AOR = 2.58, 95% CI: 1.31, 5.07), presence of diarrhea in the past two weeks (AOR = 2.05, 95% CI: 1.10, 3.85), non-exclusive breastfeeding (AOR = 2.65, 95% CI: 1.51, 4.65), and birth interval of < 24 months (AOR = 4.49, 95% CI: 2.40, 8.37) were factors significantly associated with wasting. Whereas, children in the age group of 24–59 months (AOR = 2.24, 95% CI: 1.58, 3.16), non-exclusive breastfeeding (AOR = 1.81, 95% CI: 1.24, 2.65), birth interval of fewer than 24 months (AOR = 1.54, 95% CI: 1.11, 2.14), sub-optimal child dietary diversity score (AOR = 1.59, 95% CI: 1.14, 2.22), being a non-beneficiary of productive safety-net program (AOR = 1.91, 95% CI: 1.24, 2.95), and household food insecurity (AOR = 2.60, 95% CI: 1.86, 3.64) were factors significantly associated with stunting.

**Conclusions:**

Wasting and stunting were found to be key public health problems in the South Ari District. Hence, further interventions should consider strategies to enhance household food security and integration of productive safety net programs with primary health care services. Rigorous work is required in advocating and strengthening the provision of family planning services, child care, and integrated management of common childhood illnesses. Moreover, behavioral change communication is highly demanded to improve child feeding practices.

**Supplementary Information:**

The online version contains supplementary material available at 10.1186/s40795-023-00683-3.

## Background

Adequate nutrition is a crucial part of health and development. Better nutrition is related to improved infant, child, and maternal health, stronger immune systems, safer pregnancy and childbirth, a lower risk of non-communicable diseases, and longevity [1]. Malnutrition is caused by deficiency, excess, or imbalance in the intake of energy and/or nutrients. It includes three broad conditions such as undernutrition, micronutrient-related malnutrition, and over-nutrition (overweight, obesity) [2]. Undernutrition is one form of malnutrition resulting from a lack of proper nutrition, which is necessary for growth and health [3]. It encompasses wasting (low weight for height), stunting (low height for age), and underweight (low weight for age) [2].

Under-five children are more vulnerable to malnutrition than any other age group, and their nutritional status is a sensitive indicator of their health status and nutrition [4]. A significant number of the world’s undernourished children live in countries where recurrent food insecurity and prolonged disasters occur [5]. According to the United Nations International Children’s Emergency Fund (UNICEF) conceptual framework, food insecurity is the most important proximate determinant of a child's nutritional status [6]. Food insecurity increases the risk of child malnutrition by influencing the children's food consumption and diet quality, including the quality of women's diets, as well as people's health in various ways [7]. Malnourished children are more likely to suffer from mortality and morbidity [8]. Poor nutritional status in children also contributes to poorer school enrollment, absenteeism, early dropout, and low academic achievement, all of which result in lower adult productivity [9].

Globally, the burden of child undernutrition remains high. In 2019 an estimated 21.3% or 144.0 million children and 6.9% or 47.0 million children under five are stunted and wasted, respectively. Africa and Asia bear the greatest share of all forms of malnutrition, where 54% and 40% of children are stunted, while 69% and 27% of children are wasted in Asia and Africa, respectively. Eastern Africa and southern Asia are among the regions with a very high prevalence of child stunting, at 34.5% and 31.7%, respectively [10].

Ethiopia has endorsed global and national commitments to see children free from undernutrition. Among the major commitments are the Seqota Declaration to end stunting by 2030 and Health Sector Transformation Plan to reduce childhood stunting in under five years from 40 to 26% by the end of 2020 [11]. Improving child undernutrition by using nutrition-specific and sensitive interventions is important for achieving the country’s commitment to end child undernutrition and for the achievement of sustainable development goals. Despite the above commitments, child undernutrition continues to be a major public health problem. Even if there has been a decrement in the past decade, it remains high. According to the mini-Ethiopian demographic health survey (EDHS) 2019, the prevalence of stunting, and wasting at a national level was 37% and 7%, respectively [12]. The burden of child undernutrition in the Southern Nations Nationalities and Peoples Region (SNNPR) was closest to the national prevalence, where 36.3% and 6.3% of children were stunted, and wasted, respectively [12].

Undernutrition has the greatest impact during pregnancy and early childhood—from conception to two years old, or the first 1000 days. Children who are malnourished have weakened immune systems and are thus more vulnerable to infections and illnesses [13]. Malnourished children are more likely to die from common childhood illnesses than adequately nourished children [14]. On average, a child dies every 5 seconds as a direct or indirect result of malnutrition, 700 every hour, 16,000 each day, and 6 million every year [15]. About half of infant and child deaths, an eight percent reduction in the country’s workforce, and around 16 percent of all primary school repetitions, are attributable to stunting and other forms of undernutrition [16, 17]. According to a UNICEF report, early childhood stunting is associated with a 0.7-grade loss in schooling, a 7-month delay in starting school, and a 22 to 45 percent reduction in lifetime earnings [18]. The cost of hunger report by the African Union Commission also indicated that Ethiopia loses 16.5% of its gross domestic product each year due to the long-term effects on the labor force [17].

The South Omo Zone has pastoralist, semi-pastoralist, and agrarian populations. The South Ari district is one of the agrarian districts in the South Omo Zone with a high reported number of undernourished children despite the presence of high fruit and vegetable production. The area is also known for its high cash crop production as compared to the pastoralist and semi-pastoralist districts of the zone. However, there is a dearth of evidence that tries to assess the prevalence and main predictors responsible for wasting and stunting in the district. Therefore, this study aimed to assess child undernutrition (wasting and stunting) and associated factors among children aged 06–59 months in South Ari District.

## Methods

### Study design, period, and setting

A community-based cross-sectional study was conducted from March 11 to April 11, 2021, in selected kebeles of the South Ari district. The district is located 767 kilometers (KM) from Addis Ababa, the country’s capital, 567 KM from Hawassa, the regional capital, 267 KM from Arba Minch, and 17 KM from Jinka, the zonal capital. There are 31 kebeles in the district, of which 6 are Dega, 23 Woina dega, and 2 Kolla kebeles. Based on the 2007 Ethiopian census, the projected population of the district for 2021 is 160,896, of which 80,480 are males and 80,416 are females. The total under-five years of age population is 25,121, out of which 12,555 are females and 12,566 are males. Of the under-five population, 22,429 are in the age category of 06–59 months [19]. The district is predominantly rural and depends on agriculture for economic activity. Major crops grown in the district include cereals, pulses, fruits, cassava, sweet potato, and false banana. Major crops grown in the district include cereals, pulses, and fruits. Maize, teff, wheat, and sorghum are the dominant cereal crops grown. In the area, maize, teff, and fruits are the major cash crops [20].

### Population

All households found in the South Ari district were the source population. Those randomly sampled households in selected kebeles of the South Ari district who fulfilled the eligibility criteria were the study population. All households with at least one child aged 06–59 months and who have been living in the study area for at least 06 months were included in the study. Children who were assessed and classified as having severe acute malnutrition or those who were under the therapeutic feeding program and mothers/caregivers who were not able to respond to the interview during data collection due to illness were excluded from the study.

### Sample size determination and sampling procedure

The sample size was calculated from the prevalence of stunting by using the single population proportion formula. The assumptions to be considered during the determination of the sample size were: 33.5% prevalence of stunting in the Shey Bench district, Southern Ethiopia [21], 95% confidence level, and a 5% margin of error. After considering the design effect of two and a 5% non-response rate, the largest sample size of 717 was taken for conducting the study.

**Fig. 1:**
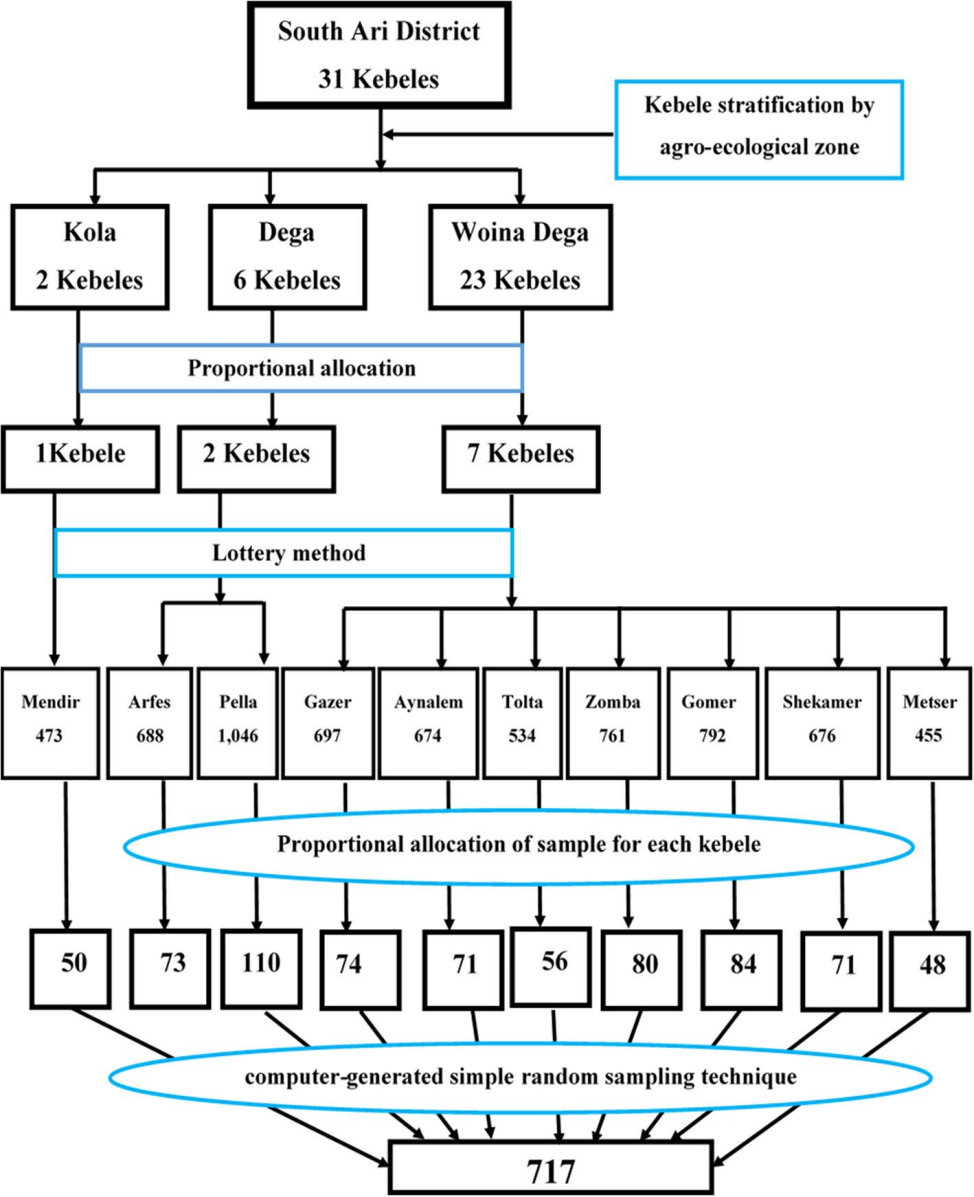
Schematic presentation for selecting study participants for a study assessing factors associated with wasting and stunting among children aged 6–59 months in South Ari District, Southern Ethiopia, 2021

A multi-stage sampling technique was used to select study participants. First, from a total of 31 kebeles found in the South Ari district, 10 kebeles were selected for the study using the lottery method. This was followed by a computer-generated simple random sampling of study households from the 10 selected study kebeles. For this purpose, a sampling frame of households eligible for the study was prepared for each kebele and entered into SPSS Version 25.0 software for random selection, and selected using SPSS select case procedure. Information about each household with children aged 06–59 months old were obtained from the health post family folder. The number of households to be included in the study from each kebele was decided using a proportional allocation based on the total number of eligible households living in the kebeles. When more than one eligible child is living in a study household, only one child is selected using the lottery method (Fig. 1).

### Variables

Stunting and wasting among children aged 06–59 months were dependent variables. Socio-demographic and economic factors such as child age, sex, maternal age, family size, marital status, household wealth index, food security status, productive safety net program beneficiary status, mother’s employment status, and mother’s educational status; maternal factors such as antenatal care follow up, place of delivery, mother’s autonomy, maternal knowledge on child feeding, and maternal dietary diversity score; child characteristics such as time of breastfeeding initiation, pre-lacteal feeding practice, complementary feeding initiation age, bottle feeding, child dietary diversity score, colostrum feeding, non-exclusive breastfeeding, immunization, birth interval, birth order, history of common childhood illness (fever, diarrhea, cough); and environmental factors such as toilet facility, source of drinking water, and hand-washing practice were independent variables.

### Data collection tool and procedures

A structured questionnaire and record form were designed to collect data on socio-demographic, economic, environmental, child and maternal characteristics, and anthropometric measurements. It was developed first in English and then translated into Amharic. Ten nurses who have exposure to under five outpatient departments and one health extension worker from each kebele conducted interviews and carried out anthropometric measurements, and two health officers supervised the overall data collection process.

Household food insecurity was measured using the Household Food Insecurity Access Scale (HFIAS) [22]. The mothers were asked nine questions related to the household’s experience of food within the 30 days preceding the survey. These questions were captured under three main domains of household food insecurity: (1) anxiety and uncertainty about food access (1 question), (2) insufficient food quality (3 questions), and (3) insufficient food intake and its physical consequences (5 questions). From these questions, a household food insecurity status as a binary outcome of food security or food insecurity was constructed.

The weight of children was measured by a portable Seca digital balance (Seca model 872, Hamburg, Germany) to the nearest 0.1 kg with light clothing and no shoes. The weight of younger children (06–23 months of age) was measured by measuring the child together with the mother, then measuring the mother alone, and finally subtracting the mother's weight from the overall weight to get the child's weight by applying the tare function. The instrument was calibrated before weighing each child by setting it to zero. Furthermore, the weighing scale was checked daily using an item with a known weight for accuracy. The length of younger children (aged 06–23 months) was measured using a standard wooden portable board in a recumbent position, and length will be recorded to the nearest 0.1 cm. Height was measured in a standing position for those children aged 24–59 months following the standard procedure. The occipital (back of the head), shoulder blades, buttocks, and heels touched the measuring board, and height was recorded to the nearest 0.1 cm. Nutritional edema was assessed by pressing the thumbs down on both feet, holding for three seconds, and observing if any bilateral indentation remained.

### Operational definitions

Stunting: refers to a low height for age. The child was classified as stunted if his/her z score was less than − 2SD; based on the WHO 2006 Child Growth Standards for the same age and sex [23]. Wasting: refers to low weight for height. The child was classified as wasted if his/her z score was less than − 2SD; based on the WHO 2006 Child Growth Standards for the same age and sex [23]. Child's dietary diversity score: was considered optimal if the child received foods made from four or more groups, and considered sub-optimal if it is less than four food groups out of the seven food groups during the previous day [24]. Diarrhea: A passage of three or more loose or liquid stools per day [25]. Information on childhood diarrhea was obtained from the mother, whether the child had experienced diarrhea symptoms two weeks before the interview. Immunization status: Children who were up to date and fully vaccinated were considered vaccinated, while those who were not vaccinated at all and defaulted were considered unvaccinated.

Exclusive breastfeeding (0–6) months: Breast milk from mother or expressed breast milk, no other liquids or solids except vitamin drops or syrups, mineral supplements, or prescribed medicines during the first six months of life [26]. Early initiation of breastfeeding: The proportion of children who were put to the breast within one hour of birth [26]. Timely initiation of complementary feeding: The proportion of children who started complementary feedings at 6 months of life [26].

Low level of maternal autonomy: from the four composite variables adapted from the demographic health survey tool, mothers who have a sum value less than the median value [27, 28]: The first three questions were related to ‘mobility’, asking the mother if she required approval from her husband or family member to go to ‘outside home’, or ‘marketplace’, or ‘health institution’. The next three questions were related to ‘mother involvement in decision making regarding her child’; specifically, ‘when a child got sick’, or ‘child schooling’ or ‘to whom to marry’. The third group of questions related to ‘financial autonomy’ inquiring about mothers' autonomy in ‘purchase of food’ or ‘big items such as oxen, land, and house’. Also, a single item on the autonomy of family planning service utilization was asked. Maternal dietary diversity score: A mother was considered to have a high dietary diversity score if she consumed at least five out of ten defined food groups the previous day or night [29]. Maternal knowledge of child feeding: was assessed by questions containing twelve items that had a yes or no response. For each correct response, a score of one, and for incorrect responses, zero was given. Mothers who scored above six (above average) were leveled as having “sufficient knowledge”, whereas, mothers who scored six and below were leveled as having “insufficient knowledge” [30, 31].

Source of water supply: Based on the EDHS category, water sources were recorded as either improved or unimproved. Piped water, protected dug wells, and springs were considered improved water sources, whereas unprotected spring/well and surface water (river, pond) were considered unimproved water sources [27]. Wealth Index: was a composite measure of the cumulative living standard of a household. The wealth index was calculated using easy-to-collect data on a household’s ownership of selected 26 types of assets [27, 32]. It was generated with a statistical procedure known as principal components analysis (PCA), the wealth index places individual households on a continuous scale of relative wealth. Each household asset was assigned a weight or factor score generated through PCA. The resulting asset scores were standardized to a standard normal distribution with a mean of zero and a standard deviation of one. These standardized scores were then used to create the breakpoints that define the wealth index as poor, medium, and rich.

### Data quality assurance

A structured questionnaire was prepared initially in English and translated, and then it was back-translated to English by different translators to check for any inconsistencies during translation. Two days of training with a practical demonstration of anthropometric measurements were given to data collectors and supervisors. To minimize anthropometric measurement errors, technical error of measurement (TEM) was computed using Emergency Nutrition Assessment (ENA) software. For computing TEM, supervisors took two weight and height measurements of ten children and let the data collectors take the measurements of all ten children twice. A pre-test on 5% of the sample was done in the Bena Tsemay district and, based on the findings, possible corrections were made. Supervision was carried out daily. At the beginning of the daily measurement session and after weighing each child, the weight scale was calibrated by setting it to zero, and it was checked daily for accuracy by using an item with a known weight. Daily, the questionnaires were checked for completeness and consistency.

### Data processing and analysis

Following data collection, data were checked and entered into Epi-Data version 3.1 before being exported to SPSS version 25.0 for data cleaning and analysis. Descriptive statistics were computed for all variables according to type. For continuous variables, mean/median, and standard deviation/interquartile range were produced, while categorical variables were assessed by computing frequencies and proportions. After checking the assumptions, the wealth index was computed by using principal component analysis and ranked into tertile.

Anthropometric measurements were converted to height-for-age Z score (HAZ) and weight-for-height Z score (WHZ) using the WHO 2006 Child Growth Standards using WHO Anthro software version 3.2.2. A binary logistic regression model was used to determine the significant association between dependent and independent variables. Crude Odds Ratios (COR) along with 95% confidence interval (CI) were used to present the results of the bivariable analysis. All variables with a significant association in bivariable analysis at *p*-value < 0.25 were entered into a multivariable logistic regression model to assess the adjusted association between dependent and independent variables. A stepwise backward likelihood ratio method was used to fit a multivariable logistic regression model to identify factors remaining in the final multivariable regression models. The adjusted odds ratio (AOR) along with a 95% confidence interval (CI) was used to determine the strength of the association. A *P*-value < 0.05 was used to declare statistical significance in the final model. Multicollinearity between independent variables was checked for all candidate variables by using a variance inflation factor (VIF). The highest observed VIF-value from both models was 2.65 (tolerance = 0.38), indicating no threat of multicollinearity. The Hosmer–Lemeshow goodness-of-fit statistic was used to check model fitness for both models and was satisfied (*p*-value ≥ 0.05).

## Result

### Socio-demographic and economic characteristics

A total of 717 respondents were successfully interviewed with a response rate of 100%. Out of the total children, 385 (53.7%) were males and 499 (69.6%) were aged 24–59 months. The majority of the mothers, 624 (87%), were married, and most of them, 525 (73.2%), were protestant. From the participants, 300 (41.8%) of the mothers had no formal education and 650 (90.6%) were Ari in ethnicity. Regarding maternal occupation, 317 (44.2%), were farmers, and 241 (33.6%) of the mothers were in the age group of 25–29 years. Of the respondents, ninety (12.6%) had a family size of eight and above. Regarding the household wealth index, 239 (33.3%) children were from poor families. Out of the study respondents, 321 (44.8%) and 594 (82.8%) children were from food insecure and productive safety-net program non-beneficiary households (Table 1).

**Table 1:** Socio-demographic and economic characteristics of children aged 06–59 months in South Ari District, Southern Ethiopia, 2021 (*N* = 717)

Variables	Categories	Frequency (N)	Percent (%)
Child Age	06–23 months	218	30.4
	24–59 months	499	69.6
Sex of child	Male	385	53.7
	Female	332	46.3
Maternal Age (in years)	15–19	2	0.3
	20–24	151	21.1
	25–29	241	33.6
	30–34	140	19.5
	≥ 35	183	25.5
Marital status	Single	70	9.8
	Married	624	87.0
	Widowed	8	1.1
	Divorced	15	2.1
Religion	Orthodox	171	23.8
	Protestant	525	73.2
	Muslim	10	1.4
	Catholic	7	1.0
	Others	6	0.6
Ethnicity	Ari	650	90.6
	Amhara	63	8.8
	Others	4	0.6
Maternal education	No formal education	300	41.8
	Primary education	286	39.9
	Secondary education and above	131	18.3
Maternal occupation	Farmer	317	44.2
	Government employee	278	38.8
	Daily laborer	34	4.7
	Merchant	51	7.1
	No work	30	4.2
	Others	7	1.0
Family size	2–4	309	43.0
	5–7	318	44.4
	≥ 8	90	12.6
Household wealth index	Poor	239	33.3
	Medium	261	36.4
	Rich	217	30.3
Household food security status	Food secure	396	55.2
	Food insecure	321	44.8
Productive safety-net program beneficiary status	Yes	123	17.2
	No	594	82.8

### Maternal and child nutrition-related characteristics

Nearly eighty percent of the mothers had four and more antenatal care (ANC) follow-ups, and a majority of them, 654 (91.2%), delivered their babies at a health facility. The majority of the mothers, 616 (85.9%) and 613 (85.5%) had a high level of maternal autonomy and sufficient maternal knowledge of child feeding practice, respectively. Almost three-fourths (532) of the participants had a high maternal dietary diversity score. Regarding common childhood illnesses, 136 (19.0%), 128 (17.9%), and 112 (15.6%) of the children had cough, diarrhea, and fever in the past two weeks preceding the study, respectively. The majority of the children, 660 (92.1%) and 684 (95.4%), initiated breastfeeding early and fed colostrum, respectively. Nearly one out of six (16.5%) children were fed pre-lacteal feeding and 200 (27.9%) were fed non-exclusive breastfeeding. Most of the children, 556 (77.7%), initiated complementary feeding at the age of 6 months, and 238 (33.2%) of the children were currently breastfeeding. Almost half of the mothers (351) use a bottle to feed their children. The majority of the children, 659 (91.9%), were immunized. More than half, 384 (53.6%), of the children had a birth interval of 24 months and above, and more than one-fourth, 193 (26.9%), of the children had a birth order of 4 and above. Regarding the child's dietary diversity score (DDS), 307 (42.8%) of the children had a sub-optimal DDS (Table 2).

**Table 2:** Maternal and child nutrition-related characteristics in South Ari District, Southern Ethiopia, 2021 (*N* = 717)

Variables	Categories	Frequency (N)	Percent (%)
ANC follow-up	No	30	4.2
	1–3	116	16.2
	≥ 4	571	79.6
Place of delivery	Home	63	8.8
	Health institution	654	91.2
Maternal autonomy	Low autonomy	101	14.1
	High autonomy	616	85.9
Maternal knowledge of child feeding practice	Sufficient	613	85.5
	Insufficient	104	14.5
Maternal dietary diversity score (DDS)	High DDS	532	74.2
	Low DDS	185	25.8
Cough in the past two weeks	Yes	136	19.0
	No	581	81.0
Diarrhea in the past two weeks	Yes	128	17.9
	No	589	82.1
Fever in the past two weeks	Yes	112	15.6
	No	605	84.4
Early initiation of breastfeeding	Yes	660	92.1
	No	57	7.9
Colostrum feeding	Yes	684	95.4
	No	33	4.6
Pre-lacteal feeding	Yes	118	16.5
	No	599	83.5
Non-exclusive breastfeeding	Yes	200	27.9
	No	517	72.1
Currently on breastfeeding	Yes	238	33.2
	No	479	66.8
Age at initiation of complementary feeding (*n* = 716)	Before 6 months	48	6.7
	At 6 months	556	77.7
	After 6 month	112	15.6
Materials used for feeding (*n* = 716)	Bottle	351	49.0
	Cup	181	25.3
	Spoon	135	18.9
	Others	49	6.8
Immunization status	Vaccinated	659	91.9
	Unvaccinated	58	8.1
Birth interval	< 24 months	333	46.4
	≥ 24 months	384	53.6
Birth order	First	220	30.7
	2–3	304	42.4
	4 and above	193	26.9
Child dietary diversity score (DDS)	Optimal	410	57.2
	Sub-optimal	307	42.8

### Environmental characteristics of study participants

Nearly half (340) of the households had an unimproved water source, and most of the households, 496 (69.2%), did not treat drinking water to make it safer. A latrine was available in a majority of the households, 657 (91.6%), and 363 (55.3%), used ventilated improved pit latrines (VIP). The majority of the respondents wash their hands after using the toilet (90.8%), before preparing food (79.6%), and before serving food (69.3%) (Table 3).

**Table 3:** Environmental characteristics of study participants in South Ari District, Southern Ethiopia, 2021 (*N* = 717)

Variables	Categories	Frequency (N)	Percent (%)
Water Source	Improved	377	52.6
	Unimproved	340	47.4
Treating drinking water to make safer	Yes	221	30.8
	No	496	69.2
Methods used to treat drinking water (*n* = 221)	Chlorination or Wuha Agar	144	65.2
	Boiling	18	8.1
	Filtering	59	26.7
Availability of latrine	Yes	657	91.6
	No	60	8.4
Types of the latrine (*n* = 657)	Flush toilet	2	0.3
	Pit latrine	292	44.4
	VIP	363	55.3
Hand washing practices after latrine use	Yes	651	90.8
	No	66	9.2
Hand washing practices before preparing food	Yes	571	79.6
	No	146	20.4
Hand washing practices before serving food	Yes	497	69.3
	No	220	30.7
Modality of hand washing practices	Always with water and soap	72	10.0
	Always with only water	69	9.6
	Always with water, and sometimes with water and soap	529	73.8
	Sometimes eat without washing my hands	47	6.6

### Undernutrition among children aged 06–59 months in South Ari District

The prevalence of wasting among children aged 06–59 months in the South Ari District was 9.10% (95% CI: 7.07%, 11.41%). Whereas, this study revealed that 59.97% (95% CI: 56.28%, 63.58%) of children aged 6–59 months in the South Ari District were stunted.

### Factors associated with wasting among children aged 06–59 months

The odds of wasting were nearly three-fold higher among children from larger family sizes (8 and above) as compared to children from smaller family sizes (2 to 4) (AOR = 3.03, 95% CI: 1.31, 7.03). Similarly, the odds of wasting were two times greater among children from larger family sizes (5 to 7) as compared to children from smaller family sizes (2 to 4) (AOR = 2.05, 95% CI: 1.11, 3.81). The odds of wasting were 3.69 times greater among children from poor families as compared to those from rich families (AOR = 3.69, 95% CI: 1.65, 8.26). The odds of wasting were 2.29 times greater among children of medium household wealth status than those of rich household wealth status (AOR = 2.29, 95% CI: 1.01, 5.16). The odds of wasting were 2.58 times higher among children of mothers with insufficient knowledge of child feeding practices than among their counterparts (AOR = 2.58, 95% CI: 1.31, 5.07). The odds of wasting were nearly two-fold increased among children with a history of diarrhea in the past two weeks as compared to their counterparts (AOR = 2.05, 95% CI: 1.10, 3.85). The odds of wasting were 2.65 times greater among children who breastfeed non-exclusively in the first 6 months of life as compared to those who breastfeed exclusively (AOR = 2.65, 95% CI: 1.51, 4.65). The odds of wasting were 4.49 times higher among children with a birth interval of fewer than 24 months as compared to children with a birth interval of 24 months and above (AOR = 4.49, 95% CI: 2.40, 8.37) (Table 4).

**Table 4:** Factors associated with wasting among children aged 06–59 months in South Ari District, Southern Ethiopia, 2021 (*N* = 717)

Variables	Wasting status		COR (95% CI)	*P*-value	AOR (95% CI)	*P*-value
	Wasted	Not wasted				
Productive safety-net program beneficiary status						
No	59 (9.9)	535 (90.1)	2.15 0.91, 5.10)	0.082	1.96 (0.79, 4.88)	0.15
Yes	6 (4.9)	117 (95.1)	1		1	
Household food security status						
Food insecure	38 (11.8)	283 (88.2)	1.84 (1.09, 3.08)	0.021	1.48 (0.83, 2.66)	0.19
Food secure	27 (6.8)	369 (93.2)	1		1	
Maternal education						
No formal education	36 (12.0)	264 (88.0)	3.44 (1.32, 8.97)	0.012	2.55 (0.89, 7.33)	0.082
Primary education	24 (8.4)	262 (91.6)	2.31 (0.86, 6.19)	0.097	1.99 (0.69, 5.73)	0.20
Secondary education & above	5 (3.8)	126 (96.2)	1		1	
Family size						
2-4	25 (8.1)	284 (91.9)	1		1	
5-7	29 (9.1)	289 (90.9)	1.14 (0.65, 1.99)	0.65	2.05 (1.11, 3.81)	0.023
≥8	11 (12.2)	79 (87.8)	1.58 (0.75, 3.35)	0.23	3.03 (1.31, 7.03)	0.01
Household Wealth Index						
Poor	32 (13.4)	207 (86.6)	3.57 (1.66, 7.67)	0.001	3.69 (1.65, 8.26)	0.001
Medium	24 (9.2)	237 (90.8)	2.34 (1.06, 5.15)	0.035	2.29 (1.01, 5.16)	0.047
Rich	9 (4.1)	208 (95.9)	1		1	
Antenatal care follow-up						
No	6 (20.0)	24 (80.0)	2.85 (1.11, 7.33)	0.029	2.28 (0.80, 6.54)	0.12
1-3	13 (11.2)	103 (88.8)	1.44 (0.75, 2.76)	0.27	1.03 (0.49, 2.14)	0.94
≥4	46 (8.1)	525 (91.9)	1		1	
Hand washing after latrine use						
Yes	56 (8.6)	595 (91.4)	1		1	
No	9 (13.6)	57 (86.4)	1.68 (0.79, 3.57)	0.18	1.34 (0.56, 3.20)	0.52
Hand washing before preparing food						
Yes	47 (8.2)	524 (91.8)	1		1	
No	18 (12.3)	128 (87.7)	1.57 (0.88, 2.79)	0.13	1.12 (0.56, 2.22)	0.75
Maternal autonomy						
Low autonomy	14 (13.9)	87 (86.1)	1.78 (0.95, 3.36)	0.07	1.21 (0.59, 2.51)	0.60
High autonomy	51 (8.3)	565 (91.7)	1		1	
Maternal knowledge of child feeding						
Insufficient	15 (14.4)	89 (85.6)	1.90 (1.02, 3.52)	0.04	2.58 (1.31, 5.07)	0.006
Sufficient	50 (8.2)	563 (91.8)	1		1	
Diarrhea in the past two weeks						
Yes	20 (15.6)	108 (84.4)	2.24 (1.27, 3.94)	0.005	2.05 (1.10, 3.85)	0.025
No	45 (7.6)	544 (92.4)	1		1	
Pre-lacteal feeding						
Yes	15 (12.7)	103 (87.3)	1.60 (0.87, 2.96)	0.13	1.70 (0.78, 3.73)	0.18
No	50 (8.3)	549 (91.7)	1		1	
Non-exclusive breastfeeding						
Yes	28 (14.0)	172 (86.0)	2.11 (1.25, 3.56)	0.005	2.65 (1.51, 4.65)	0.001
No	37 (7.2)	480 (92.8)	1		1	
Currently breastfeeding						
Yes	16 (6.7)	222 (93.3)	1		1	
No	49 (10.2)	430 (89.8)	1.58 (0.88, 2.84)	0.13	1.47 (0.76, 2.83)	0.25
Immunization status						
Unvaccinated	8 (13.8)	50 (86.2)	1.69 (0.76, 3.74)	0.20	1.22 (0.49, 3.02)	0.67
Vaccinated	57 (8.6)	602 (91.4)	1		1	
Birth interval						
<24 months	46 (13.8)	287 (86.2)	3.08 (1.77, 5.37)	<0.001	4.49 (2.40, 8.37)	<0.001
≥24 months	19 (4.9)	365 (95.1)	1		1	
Child dietary diversity score (DDS)						
Sub-optimal	33 (10.7)	274 (89.3)	1.42 (0.85, 2.37)	0.18	1.04 (0.58, 1.88)	0.88
Optimal	32 (7.8)	378 (92.2)	1		1	
Water Source						
Improved	27 (7.2)	350 (92.8)	1		1	
Unimproved	38 (11.2)	302 (88.8)	1.63 (0.97, 2.74)	0.064	0.80 (0.43, 1.50)	0.50
Treating drinking water						
Yes	12 (5.4)	209 (94.6)	1		1	
No	53 (10.7)	443 (89.3)	2.08 (1.09, 3.98)	0.026	1.61 (0.79, 3.28)	0.19

NB: 1= Reference category; Hosmer and Lemeshow Test (*P*-value= 0.11)

### Factors associated with stunting among children aged 06–59 months

The odds of being stunted were 2.24 times greater among children in the age group of 24–59 months as compared to children in the age group of 6–23 months (AOR = 2.24, 95% CI: 1.58, 3.16). The odds of being stunted were 1.81 times higher among children who breastfeed non-exclusively than among those who breastfeed exclusively (AOR = 1.81, 95% CI: 1.24, 2.65). The odds of stunting were 1.54 times increased among children with a birth interval of fewer than 24 months as compared to children with a birth interval of 24 months and above (AOR = 1.54, 95% CI: 1.11, 2.14). The odds of being stunted were 1.59 times greater among children who had sub-optimal child dietary diversity scores as compared to their complements (AOR = 1.59, 95% CI: 1.14, 2.22). The odds of stunting were nearly two-fold higher among children from productive safety net program non-beneficiary households compared to their counterparts (AOR = 1.91, 95% CI: 1.24, 2.95). The odds of stunting were 2.6 times greater among children from food-insecure households as compared to those from food-secure households (AOR = 2.60, 95% CI: 1.86, 3.64) (Table 5).

**Table 5:** Factors associated with stunting among children aged 06–59 months in South Ari District, Southern Ethiopia, 2021 (*N* = 717)

Variables	Stunting status		COR (95% CI)	*P*-value	AOR (95% CI)	*P*-value
	Stunted	Not stunted				
Child Age						
6-23 months	101 (46.3)	117 (53.7)	1		1	
24-59 months	329 (65.9)	170 (34.1)	2.24 (1.62, 3.10)	<0.001	2.24 (1.58, 3.16)	<0.001
Sex of child						
Male	222 (57.7)	163 (42.3)	0.81 (0.60, 1.10)	0.17	0.79 (0.57, 1.09)	0.15
Female	208 (62.7)	124 (37.3)	1		1	
Household wealth index						
Poor	149 (62.3)	90 (37.7)	1.27 (0.87, 1.84)	0.22	0.74 (0.48, 1.13)	0.16
Medium	158 (60.5)	103 (39.5)	1.17 (0.81, 1.69)	0.39	0.94 (0.63, 1.40)	0.75
Rich	123 (56.7)	94 (43.3)	1		1	
Hand washing after latrine use						
Yes	384 (59.0)	267 (41.0)	1		1	
No	46 (69.7)	20 (30.3)	1.60 (0.93, 2.77)	0.093	0.82 (0.46, 1.47)	0.497
Hand washing before preparing food						
Yes	333 (58.3)	238 (41.7)	1		1	
No	97 (66.4)	49 (33.6)	1.42 (0.97, 2.07)	0.075	0.81 (0.53, 1.23)	0.32
Maternal knowledge of child feeding practice						
Insufficient	69 (66.3)	35 (33.7)	1.38 (0.90, 2.13)	0.15	1.23 (0.76, 1.99)	0.397
Sufficient	361 (58.9)	252 (41.1)	1		1	
Maternal dietary diversity score (DDS)						
Low DDS	126 (68.1)	59 (31.9)	1.60 (1.13, 2.28)	0.009	1.03 (0.67, 1.58)	0.90
High DDS	304 (57.1)	228 (42.9)	1		1	
Diarrhea in the past two weeks						
Yes	87 (68.0)	41 (32.0)	1.52 (1.01, 2.28)	0.043	1.26 (0.81, 1.96)	0.296
No	343 (58.2)	246 (41.8)	1		1	
Non-exclusive breastfeeding						
Yes	131 (65.5)	69 (34.5)	1.38 (0.99, 1.94)	0.06	1.81 (1.24, 2.65)	0.002
No	299 (57.8)	218 (41.2)	1		1	
Currently breastfeeding						
Yes	109 (45.8)	129 (54.2)	1		1	
No	321 (67.0)	158 (33.0)	2.40 (1.75, 3.31)	<0.001	1.55 (0.90, 2.65)	0.11
Birth interval						
<24 months	213 (64.0)	120 (36.0)	1.37 (1.01, 1.85)	0.042	1.54 (1.11, 2.14)	0.011
≥24 months	217 (56.5)	167 (43.5)	1		1	
Child dietary diversity score (DDS)						
Sub-optimal	205 (66.8)	102 (33.2)	1.65 (1.22, 2.25)	0.001	1.59 (1.14, 2.22)	0.006
Optimal	225 (54.9)	185 (45.1)	1		1	
Water source						
Improved	207 (54.9)	170 (45.1)	1		1	
Unimproved	223 (65.6)	117 (34.4)	1.57 (1.16, 2.12)	0.004	1.09 (0.77, 1.56)	0.62
Productive safety-net program beneficiary status						
No	379 (63.8)	215 (36.2)	2.49 (1.68, 3.70)	<0.001	1.91 (1.24, 2.95)	0.003
Yes	51 (41.5)	72 (58.5)	1		1	
Household food security status						
Food insecure	236 (73.5)	85 (26.5)	2.89 (2.11, 3.97)	<0.001	2.60 (1.86, 3.64)	<0.001
Food secure	194 (49.0)	202 (51.0)	1		1	

NB: 1= Reference category; Hosmer and Lemeshow Test (*P*-value=0.297)

## Discussion

This study aimed to assess factors associated with wasting and stunting among children aged 6–59 months in South Ari District. In this study, 9.10% and 59.97% of children aged 6–59 months were wasted and stunted, respectively. Having a larger family size, poor household wealth index, insufficient maternal knowledge on child feeding practice, history of diarrhea, non-exclusive breastfeeding, and shorter birth interval were significant factors associated with wasting. While older age of a child, non-exclusive breastfeeding, shorter birth interval, sub-optimal child dietary diversity score, not using productive safety-net program service, and household food insecurity were factors significantly associated with stunting.

In this study, the prevalence of wasting among children aged 06–59 months was 9.10%. This finding is comparable with findings reported from Kenya 8%, and different parts of Ethiopia 6.8% to 13.4% [33–39]. This finding is also in line with the mini-Ethiopia Demographic Health Survey 2019 finding for the Southern region 6.3% [12]. However, this is lower than studies reported from Bangladesh (18.2%), and different parts of Ethiopia 16% to 34.6% [40–44]. This discrepancy might be explained partly due to socioeconomic differences, seasonal variation, feeding habits of the study population, and differences in study setting. This finding revealed that the prevalence of stunting was 59.97%. This finding is consistent with findings reported from different parts of Ethiopia 52.4% to 64.5% [41, 43, 45, 46]. However, this study finding is higher than studies conducted in rural Bangladesh 36.8%, rural part of Kenya 31%, and different parts of Ethiopia 42.3% to 47.6% [33–36, 40, 44]. A possible explanation for this difference might be due to a variation in the socioeconomic status, sample size, setting, agroecology, and feeding habits of the study population.

In the current study, having larger family sizes (8 and above) had increased the risk of wasting among children nearly three-fold as compared to smaller family sizes (2 to 4). Similarly, having larger family sizes (5 to 7) had nearly two-fold higher odds for wasting as compared to smaller family sizes (2 to 4). This finding is supported by studies reported from different parts of Ethiopia [40, 47]. This might be partially explained by the fact that families with large sizes were more likely to share available food among all members, and they could have increased economic constraints for a variety of food consumption. And this might affect the adequate daily intake of a balanced diet and increase children's experience of suffering from being wasted.

In this study, children from poorer families had 3.69 times greater odds of being wasted as compared to those who were from rich families. Similarly, those children from families with a medium wealth index were 2.29 times more likely to be wasted than those from families with a rich wealth index. This finding is in agreement with a study reported in rural Bangladesh [44]. Partly, this might be explained by the fact that those families with poor and medium wealth indexes have poor dietary diversity, which might increase levels of being wasted. Inversely, families with a higher wealth index and more resources tend to have more access to diverse diets and lower levels of being wasted. Rich households often use an extra income to purchase non-staple foods, thus increasing household dietary diversity and which might reduce being wasted in their children. Hence, further interventions for improving socio-economic status are highly demanding.

According to the findings of this study, children from mothers with insufficient knowledge on child feeding practice had 2.58 times higher odds of wasting than their counterparts. This might be explained by the fact that mothers with insufficient knowledge of child feeding practices were less likely to implement feeding recommendations for children before and during illness. This may lead to inadequate nutrient intake and negatively affect nutritional status. Moreover, mothers with insufficient knowledge of child feeding practices might experience unhygienic practices when preparing food and feeding their children. This might increase the risk of infection and being wasted. This finding is also supported by current findings, in which nearly 42% of the mothers have no formal education.

In this study, children with a history of diarrhea in the past two weeks were nearly two-fold more likely to be wasted as compared to their counterparts. This finding is in line with other studies reported from different parts of Ethiopia [34, 37, 40]. This might be partially explained by the fact that diarrhea may lead to loss of appetite, decreased dietary intake, poor digestion, and malabsorption of nutrients, which finally may result in wasting. This might imply the vicious cycle of infection and malnutrition. However, it is difficult to conclude the temporal relationship because of the nature cross-sectional study.

In the current study, children who were not exclusively breastfed in the first six months of life had a 2.65 times greater chance of being wasted than their counterparts. This finding is in line with finding reported from Gonder [35]. A possible reason for this might be that the initiation of complementary foods in the first 06 months may affect optimal breastfeeding practice and reduce the intake of essential nutrients from breast milk needed for optimal growth, and might end up being wasted. Moreover, early initiation of complementary feeding might increase the chance of infection as there might be poor hygienic practices. This may result in a loss of appetite, decreased nutrient intake, and increased energy demands.

The finding from this study attested that children with a birth interval of fewer than 24 months had 4.49 times higher odds of being wasted than those children with a birth interval of 24 months and above. This finding is supported by further analysis of the Ethiopian Demographic and Health Survey [48]. Partly, this might be because a shorter birth interval might increase food sharing among siblings and compromise the provision of quality care and breastfeeding duration of index children. Moreover, a short birth interval may result in a depleted maternal nutrient reserve, and this could increase intrauterine growth restriction and poor nutritional status because of an intergenerational link to malnutrition.

In this study, the age of the child showed a significant association with stunting, as evidenced by a higher risk of stunting in the older age group. Children in the age group of 24–59 months had more than a two-fold increased odds of stunting as compared to those children in the age group of 06–23 months. This finding is in agreement with other studies reported from different parts of Ethiopia [40, 46, 47, 49, 50]. This might be explained by the fact that stunting is chronic in nature and commonly present after a long-term nutritional shortage. This might be due to increased nutritional demand for growth and development as the child gets older. Moreover, the child might be on a family diet and may face inadequate dietary intake. Hence, the risk of diminished linear growth increases as the child becomes older.

In this study, children who breastfed non-exclusively had nearly two-fold higher odds of stunting than their counterparts. This finding is consistent with other studies reported from Ethiopia [50, 51]. Partly, this might be due to the initiation of non-exclusive breastfeeding before six months, which might increase the risk of gastrointestinal infections, which may result in nutrient depletion and stunting. Besides, non-exclusive breastfeeding may negatively affect optimal breastfeeding and can result in inadequate dietary intake.

The finding from this study revealed that children with a birth interval of < 24 months had 1.54 times higher odds of stunting as compared to those children with a birth interval of 24 months and above. This finding is supported by other studies reported from Ethiopia [21, 51]. A possible reason for this might be that a child with a shorter preceding birth interval may not breastfeed for two years, and this might negatively affect the linear growth of the child. Moreover, mothers may not have adequate time to feed and give appropriate care to their children, and this might increase stunting.

This study revealed that a child with a sub-optimal dietary diversity score had higher odds of stunting as compared to their complements. This finding is in agreement with studies reported from Myanmar and Ethiopia [52, 53]. This might be explained by the fact that children who had sub-optimal dietary diversity scores experienced inadequate intake of balanced diets needed for linear growth. Moreover, those children who face inadequate dietary intake can have impaired immunity and were at risk of infection, which might negatively affect their nutritional status.

The finding from this study showed that children living in a productive safety net program (PSNP) non-beneficiary households had nearly two-fold higher odds of stunting compared to those in PSNP beneficiary households. Partly, this might be because being a PSNP beneficiary increases income earnings for households and may result in improved access and intake of a variety of foods. This might result in improved nutritional status.

In this study, children living in food-insecure households had 2.6 times higher odds of stunting as compared to those living in food-secure households. This finding is in line with findings reported from Malaysia and Ethiopia [54, 55]. This might be explained by the fact that stunting results from a prolonged period of inadequate nutrient intake. Hence, children from households with food insecurity experience inadequate intake of nutrients in the long term, and this might cause them to suffer from stunting. Household food insecurity differs across time, and sometimes it may stay for a long period, resulting in stunting. Moreover, household food insecurity is one of the most important proximate determinants of a child's nutritional status [6]. This is also supported by the current study, in which nearly 45% of children live in food insecure households.

This finding implies that wasting and stunting continues to be the major public health problem in South Ari District, which requires consistent and integrated interventions from the regional health bureau, zonal health department, district health office, and all other concerned bodies for achieving the country’s commitment to end child undernutrition and for the achievement of sustainable development goals. The finding also implies that further nutrition-specific and sensitive interventions are needed by addressing identified factors for improving child wasting and stunting.

### Limitations of the study

The study has the following limitations. Since the study used a cross-sectional design, it was difficult to see any potential temporal (cause-effect) associations. A certain level of recall bias was expected among respondents answering questions regarding events that happened in the past; such as 24-h recall for dietary diversity, child’s history of illness, 4 weeks recall for household food security status, and breastfeeding patterns immediately after birth, and then after, since it is a relatively long period to expect people to remember. However, due attention was given to reminding the event that happened in the past by associating it with a known event, and hopefully, the recall problem was of no differential nature on the exposure status. The study might be affected by measurement error during the collection of anthropometric data even though due attention was given to the study procedures, such as the process of training, pretesting, instrument calibration, standardization of measurement, and close supervision throughout the field activities to minimize bias.

## Conclusion

Wasting and stunting were found to be key public health problems in the South Ari District. Wasting was associated with larger family size, low and medium wealth index, insufficient maternal knowledge of child feeding practice, history of diarrhea in the last two weeks, non-exclusive breastfeeding, and shorter birth interval. Whereas, older child age, non-exclusive breastfeeding, shorter birth interval, sub-optimal child dietary diversity score, non-beneficiary of a productive safety-net program, and household food insecurity were factors associated with stunting. Therefore, behavioral change communication is highly demanding in improving child feeding practice and also requires rigorous work in advocating for the provision of family planning services for child spacing. Strengthening child care and integrated management of common childhood illnesses are needed. Moreover, further intervention to enhance household food security and integration of productive safety-net programs with primary health care services is required.

## Supplementary Information

**Additional file 1.**

**Additional file 2.**

## Data Availability

All the data generated and analyzed during this study are included, in the form of tables, and texts. The whole dataset used to analyze factors associated with wasting and stunting among children aged 06–59 months in the South Ari District is attached as supplementary materials.

## Abbreviations

AOR: Adjusted Odds Ratio
COR: Crude Odds Ratio
EDHS: Ethiopian Demographic Health Survey
HAZ: Height-for-age Z-score
HFIAS: Household Food Insecurity Access Scale
PCA: Principal Component Analysis
SNNPR: Southern Nation Nationalities, and Peoples Region
SPSS: Statistical Package for Social Sciences
SSA: Sub-Sahara Africa
UNICEF: United Nations International Children’s Emergency Fund
WHZ: Weight-for-height Z-score
WHO: World Health Organization

## References

[1] WHO. Nutrition. 2020; Available from: https://www.who.int/healthtopics/nutrition, Accessed on 28 Oct 2020.

[2] Singh A. Childhood Malnutrition in India. Intech Open. 2020;13.

[3] UNICEF. Nutrition Glossary. 2012; Available from: http://wiredhealthresources.net/presentations/82/story_content/external_files/4.Nutrition_Glossary, Accessed on 02 Jan 2020.

[4] Senthilkumar SK, Chacko TV, Suvetha K (2018). Nutritional status assessment of children aged 0–5 years and its determinants in a tribal community of Coimbatore district. Int J Community Med Public Health.

[5] United Nations Children’s Fund (UNICEF). Improving Child Nutrition, The achievable imperative for global progress. 2013; Available from: http://www.unicef.org/publications/index_68661.html Accessed on 02 Jan 2020.

[6] Strategy for improved nutrition of children and women in developing countries. United Nations Children's Fund. Indian J Pediatr. 1991 Jan-Feb;58(1):13–24. DOI: 10.1007/BF02810402. PMID: 1937618. 10.1007/BF02810402 1937618

[7] FAO, IFAD, UNICEF W and W. The State of Food Security and Nutrition in the World 2020. Transforming food systems for affordable healthy diets. Rome, FAO. 2020; Available from: 10.4060/ca9692en, Accessed on 10 Nov 2020.

[8] Victora CG, Adair L, Fall C, Hallal PC, Martorell R, Richter L (2008). Maternal and child undernutrition: consequences for adult health and human capital. Lancet.

[9] Aguayo VM, Nair R, Badgaiyan N, Krishna V (2016). Determinants of stunting and poor linear growth in children under 2 years of age in India: an in-depth analysis of Maharashtra's comprehensive nutrition survey. Matern Child Nutr.

[10] United Nations Children’s Fund (UNICEF), World Health Organization IB for R and DWB. Levels and trends in child malnutrition: Key Findings of the 2020 Edition of the Joint Child Malnutrition Estimates. Geneva: World Health Organization; 2020. License: CC BY-NCSA 3.0 IGO. Available from: https://apps.who.int/iris/rest/bitstreams/1273507/retrieve, Accessed on 10 Nov 2020.

[11] FMOH. Federal Democratic Republic of Ethiopia Ministry of Health. HSTP: Health Sector Transformation Plan (2015/16–2019/20). 2015; Available from: https://www.globalfinancingfacility.org/sites/gff_new/files/Ethiopia-health-systemtransformation-plan.pdf, Accessed on 28 Oct 2020.

[12] Ethiopian Public Health Institute (EPHI) [Ethiopia] and ICF. Ethiopia Mini Demographic and Health Survey 2019: Key Indicators. Rockville, Maryland, USA: EPHI and ICF. 2019.

[13] UNICEF. Progress for children: a world fit for children statistical review. 2007; Available from: http://www.unicef.org/publications/files/Progress_for_Children_No_6_revised.pdf, Accessed on 30 Oct 2020.

[14] UNICEF. The State of World’s Children 1998: Report on Malnutrition: Causes, consequences, and solutions. 1998;56(4):115–23. Available from: https://www.unicef.org/sowc/archive/ENGLISH/TheStateoftheWorld%27sChildren1998.pdf, Accessed on 10 Nov 2020. 10.1111/j.1753-4887.1998.tb01723.x 9584496

[15] Food and Agriculture Organization of the United Nations (FAO). The State of Food Insecurity in the World: When people Live with Hunger and Fear Starvation. 2002; Available from: http://www.fao.org/docrep/pdf/005/y7352e/y7352e00.pdf, Accessed on 02 Jan 2020.

[16] African Union Commission. NEPAD Planning and coordinating Agency, UN Economic Commission of Africa, and UN World Food Programme. The Cost of Hunger in Africa: Social and Economic Impact of Child Undernutrition in Egypt, Ethiopia, Swaziland, and Uganda. Report. Addis Ababa UNECA. 2014; Available from: https://www.uneca.org/sites/default/files/uploaded-documents/CoM/com2014/com2014-the_cost_of_hunger-english.pdf, Accessed on 10 Jan 2020.

[17] OECD Development Pathways. “Assessment of the long-term financing of social protection in Ethiopia.” 2018; Available from: https://ec.europa.eu/trustfundforafrica/sites/euetfa/files/t05-eutf-hoa-et-72_-_drr_in_ethiopia.pdf, Accessed on 05 Jan 2020.

[18] UNICEF; European Union. Improving Nutrition Security in Africa an EU‐UNICEF joint action. Available from: https://www.unicef.org/french/education/files/EUUNICEF_Africa.pdf, Accessed on 23 Nov 2020.

[19] South Ari District Health Office. Health Report. 2020; Accessed on 30 Oct 2020.

[20] South Ari District Agriculture and Rural Development Office. Agricultural Report. 2020; Accessed on 30 Oct 2020.

[21] Teferi MB, Hassen HY, Kebede A, Adugnaw E, Gebrekrstos G, Guesh M (2016). Prevalence of stunting and associated factors among children aged 06–59 months in southwest ethiopia: a cross-sectional study. J Nut Health Food Sci.

[22] Coates J SA, Bilinsky P. . Household Food Insecurity Access Scale (HFIAS) for Measurement of Household Food Access: Indicator Guide (v. 3). Washington, D.C.: Food 57 | Pag e and Nutrition Technical Assistance Project, Academy for Educational Development. 2007;3(August). Available from: http://www.fao.org/fileadmin/user_upload/eufaofsi4dm/doctraining/hfias.pdf, Accessed on 02 Jan 2020.

[23] WHO. Multicentre Growth Reference Study Group. WHO Child Growth Standards: Length/height-for-age, weight-for-age, weight-for-length, weight-for-height, and body mass index-for-age: Methods and development. 2006; Available from: https://www.who.int/childgrowth/standards/technical_report/en/ Accessed on 30 Dec 2019.

[24] INDDEX Project (2018). Data4Diets: Building Blocks for Diet-related Food Security Analysis. Tufts University, Boston, MA. 2018:2006–8. Available from: https://inddex.nutrition.tufts.edu/data4diets,Accessed on 02 Jan 2020.

[25] WHO. Factsheet on diarrheal disease. 2017; Available from: https://www.who.int/newsroom/fact-sheets/detail/diarrhoeal-disease; Accessed on 06 Jan 2020.

[26] WHO. "Indicators for assessing infant and young child feeding practices: part 1: definitions: conclusions of a consensus meeting held 6–8 November 2007 in Washington DC, USA. 2007(November): 1–19, Accessed on 30 Oct 2020.

[27] Central Statistical Agency (CSA) [Ethiopia] and ICF. Ethiopia Demographic and Health Survey 2016. Addis Ababa, Ethiopia, and Rockville, Maryland, USA: CSA and ICF. Available from: https://dhsprogram.com/pubs/pdf/FR328/FR328.pdf. Accessed on 30 Dec 2019.

[28] Tosheno D, Adinew YM, Thangavel T, SB. W. Risk Factors of Underweight in Children Aged 6 – 59 Months in Ethiopia. Journal of nutrition and metabolism. 2017. 10.1155/2017/6368746 PMC5702944 29259827

[29] Nkhoma B, Ng’ambi WF, Chipimo PJ, Zambwe M. FAO and FHI 360. Minimum Dietary Diversity for Women: A Guide for Measurement. Rome: FAO. 2016; Available from: http://www.fao.org/3/a-i5486e.pdf, Accessed on 02 Jan 2020.

[30] Wu Q, Scherpbier RW, Van VMH, Chen L, Wang W, Li Y (2014). Poor infant and young child feeding practices and sources of caregivers ’ feeding knowledge in rural Hebei Province, China : findings from a cross-sectional survey. BMJ Open.

[31] Egata G, Berhane Y, Worku A (2013). Predictors of non-exclusive breastfeeding at 6 months among rural mothers in east Ethiopia : a community-based analytical cross-sectional study. Int Breastfeed J.

[32] Abate KH, Belachew T. Women’s autonomy and men’s involvement in child care and feeding as predictors of infant and young child anthropometric indices in coffee farming households of Jimma Zone, South West of Ethiopia. Renzaho AMN, editor. PLoS One. 2017 Mar 6;12(3): e0172885. 10.1371/journal.pone.0172885 PMC5338789 28264008

[33] Tankoi EoO, Asito SA, Adoka S (2016). Determinants of malnutrition among children aged 6–59 months in Trans-Mara East Sub-County, Narok County, Kenya. Int J Pub Health Safe..

[34] Asfaw M, Wondaferash M, Taha M, Dube L (2015). Prevalence of undernutrition and associated factors among children aged between six to fifty-nine months in Bule Hora district. South Ethiopia BMC Public Health.

[35] Abebe Z, Anlay DZ, Biadgo B, Kebede A, Melku T, Enawgaw B (2017). High prevalence of undernutrition among children in Gondar Town, Northwest Ethiopia: a community-based cross-sectional study. Int J Pediatr.

[36] Gelu A, Edris M, Derso T, Abebe Z (2018). Undernutrition and associated factors among children aged 6–59 months living in slum areas of Gondar City, Northwest Ethiopia: a cross-sectional study. Pediatric Health Med Ther.

[37] Tufa EG, Dake SK, Bekru ET, Tekle HA, Bobe TM, Angore BN (2018). Magnitude of wasting and underweight among children 6–59 months of age in Sodo Zuria District, South Ethiopia: a community-based cross-sectional study. BMC Res Notes.

[38] Mulu E, Mengistie B (2017). Household food insecurity and its association with nutritional status of under-five children in Sekela District, Western Ethiopia: a comparative cross-sectional study. BMC nutrition.

[39] Yisak H, Gobena T, Mesfin F (2015). Prevalence and risk factors for undernutrition among children under five at Haramaya district. Eastern Ethiopia BMC Pediatr.

[40] Gebre A, Reddy PS, Mulugeta A, Sedik Y, Kahssay M (2019). Prevalence of malnutrition and associated factors among under-five children in pastoral communities of Afar Regional State, Northeast Ethiopia: a community-based cross-sectional study. J Nutr Metab.

[41] Fentahun W, Wubshet M, Tariku A (2016). Undernutrition and associated factors among children aged 6–59 months in East Belesa District, northwest Ethiopia: a community-based cross-sectional study. BMC Public Health.

[42] Motbainor A, Worku A, Kumie A (2015). Stunting is associated with food diversity while wasting with food insecurity among under-five children in East and West Gojjam Zones of Amhara Region, Ethiopia. PLoS ONE.

[43] Alemayehu M, Tinsae F, Haileslassie K, Seid O, Gebregziabher G, Yebyo H (2015). Undernutrition status and associated factors in under-5 children, in Tigray. Northern Ethiopia Nutrition.

[44] Ali NB, Tahsina T, Hoque DME, Hasan MM, Iqbal A, Huda TM (2019). Association of food security and other socio-economic factors with dietary diversity and nutritional statuses of children aged 6–59 months in rural Bangladesh. PLoS ONE.

[45] Tariku A, Biks GA, Derso T, Wassie MM, Abebe SM (2017). Stunting and its determinant factors among children aged 6–59 months in Ethiopia. Ital J Pediatr.

[46] Abeway S, Gebremichael B, Murugan R, Assefa M, Adinew YM. Stunting and Its Determinants among Children Aged 6–59 Months in Northern Ethiopia: A Cross-Sectional Study. J Nutr Metab. 2018:1078480. 10.1155/2018/1078480 PMC6036796 30046469

[47] Geberselassie SB, Abebe SM, Melsew YA, Mutuku SM, Wassie MM (2018). Prevalence of stunting and its associated factors among children 6–59 months of age in Libo-Kemekem district, Northwest Ethiopia; a community-based cross-sectional study. PLoS ONE.

[48] Dessie ZB, Fentie M, Abebe Z, Ayele TA, Muchie KF (2019). Maternal characteristics and nutritional status among 6–59 months of children in Ethiopia: further analysis of demographic and health survey. BMC Pediatr.

[49] Moges B, Feleke A, Meseret S, Doyore F (2015). Magnitude of stunting and associated factors among 6–59 months old children in Hossana Town, Southern Ethiopia. J Clinic Res Bioeth.

[50] Moges H, Alemayehu D, Redi H, Gebeyehu Y, Dires A, Gedamu S (2019). Prevalence and associated factors of stunting among children aged six month - five year in Ataye Town, Northeast Ethiopia. Int J Nutr Food Sci.

[51] Kahssay M, Woldu E, Gebre A, Reddy S (2020). Determinants of stunting among children aged 6 to 59 months in pastoral community, Afar region, North East Ethiopia: unmatched case-control study. BMC Nutrition.

[52] Fekadu Y, Mesfin A, Haile D, Stoecker BJ (2015). Factors associated with nutritional status of infants and young children in Somali Region, Ethiopia: a cross-sectional study. BMC Public Health.

[53] Hein AK, Hong SA, Puckpinyo A, Tejativaddhana P (2019). Dietary diversity, social support and stunting among children aged 6–59 months in an internally displaced persons camp in Kayin State. Myanmar Clin Nutr Res.

[54] Betebo B, Ejajo T, Alemseged F, Massa D (2017). Household food insecurity and its association with nutritional status of children 6–59 months of age in East Badawacho District. South Ethiopia J Environ Public Health.

[55] Naser IA, Jalil RA, Wan Manan WM, Wan Suriati WN, Zalilah MS, Mohamed RA (2015). Assessment of food insecurity and nutritional outcomes in Bachok, Kelantan. J Nutr Food Sci.

