# Supplementary material for: Factors associated with wasting and stunting among children aged 06–59 months in South Ari District, Southern Ethiopia: a community-based cross-sectional study
Source: BMC Nutr. 2023 Feb 24;9:34. doi: 10.1186/s40795-023-00683-3 (PMC9950702; doi:10.1186/s40795-023-00683-3)
Supplement: Supplementary file 2 — Additional file 2. [file 40795_2023_683_MOESM2_ESM.docx]

**English version questionnaire**

**Instructions:** The respondent should be the mother/caregivers and are expected to answer issues related to the household and the selected child.

**Questionnaire code: _______**

**Section A. Socio-demographic and economic characteristics**

| **Name** | **Variables** | **Responses** | | **Skip to Q** |
| --- | --- | --- | --- | --- |
| **A1** | Identification number (ID) |  | |  |
| **A2** | Date of interview |  | |  |
| **A3** | Kebele |  | |  |
| **A4** | Total Number of household members | ______________ | |  |
| **A5** | Number of household members whose age is below 15 and above 65 | ___________________________ | |  |
| **A6** | Number of household members whose age is between 15 and 65 | ___________________________ | |  |
| **A7** | How old are you? | Age in completed years___________ | |  |
| **A8** | Current marital status | 1. Single  2. Married  3. Widowed  4. Divorced | |  |
| **A9** | Religion | 1. Orthodox  2. Protestant  3. Muslim  4. Catholic  5. Others (specify)_____________________ | |  |
| **A10** | Ethnicity | 1. Ari  2. Amhara  3. Wolaita  4. Goffa  5. Other (specify) __________ | |  |
| **A11** | Educational level of the mother | 1. No formal education 2. Primary education 3. Secondary education 4. College/University | |  |
| **A12** | Educational level of the husband | 1. No formal education 2. Primary education 3. Secondary education 4. College/University | |  |
| **A13** | Occupation of the Father | 1. Farmer  2. Government employee  3. Daily laborer  4. Merchant  5. No work  6. Other (specify)___________ | |  |
| **A14** | Occupation of the mother | 1. Housewife  2. Farmer  3. Government employee  4. Daily laborer  5. Merchant  6. Other (specify)___________ | |  |
| **Household Assets** | | | | |
| ***Now I will ask you about some fixed assets that your household has*.** | | | | |
| Does the household have any of the following properties? **(Circle)** | | | **Yes** | **No** |
| **A15** | Functioning radio/Tape recorder/CD player | | 1 | 0 |
| **A16** | Functioning Television | | 1 | 0 |
| **A17** | Gas Stove | | 1 | 0 |
| **A18** | Kerosene stove | | 1 | 0 |
| **A19** | Electric stove | | 1 | 0 |
| **A20** | Bicycle | | 1 | 0 |
| **A21** | Motor Cycle | | 1 | 0 |
| **A22** | Cart/Gari | | 1 | 0 |
| **A23** | Watch (Hand/Wall) | | 1 | 0 |
| **A24** | Mobile phone | | 1 | 0 |
| **A25** | Plough | | 1 | 0 |
| **A26** | Sofa | | 1 | 0 |
| **A27** | Spring mattress | | 1 | 0 |
| **A28** | Sponge/Foam mattress | | 1 | 0 |
| **A29** | Cotton mattress | | 1 | 0 |
| **A30** | Grass Mattress | | 1 | 0 |
| **A31** | Chair/Stool | | 1 | 0 |
| **A32** | Generator | | 1 | 0 |
| **A33** | Milling | | 1 | 0 |
| **A34** | Water pump | | 1 | 0 |

**Section B. Environmental conditions**

| **Name** | **Variable** | **Response** | **Skip** |
| --- | --- | --- | --- |
| **B1** | What is the major source of water supply for drinking? | 1. Pipe 2. Protected spring/ well 3. Unprotected spring/well 4. Surface water (river, pond, and stream) 5. Other (specify) _________ |  |
| **B2** | Do you treat the water to make it safer for drinking? | 1. Yes 0. No | If No to B4 |
| **B3** | If YES to B2, What method do you usually use to make water safer for a drink? | 1. Chlorination/ Wuha Agar 2. Boiling 3. Filtering 4. Other, specify_____________ |  |
| **B4** | Do you have a latrine? | 1. Yes 0. No | If No to C1 |
| **B5** | Type of latrine you use? (Observation) | 1. Flush toilet 2. Pit latrine 3. VIP 4. Other (specify)__________ |  |

**Section C. Child characteristics**

| **Name** | **Variable** | **Response** | **Skip** |
| --- | --- | --- | --- |
| **C1** | What is the Sex of a Child? | 1. Male  2. Female |  |
| **C2** | What is the child's date of birth?  *(Confirm child’s date of birth by looking Family Health card or vaccination card, or local calendar of events)* | _______/_______/______________ |  |
| **C3** | Age of the child in completed months | ________________________________ |  |
| **C4** | Place of delivery of the child | 1. Home  2. Health institution |  |
| **C5** | Immunization status of the child  *(Confirm by looking at child’s Immunization card)* | 1. Not vaccinated at all 2. Up to date (on appointment)   3. Defaulted  4. Fully vaccinated |  |
| **C6** | What is the birth order of the child? | _____________________ |  |
| **C7** | What is the birth interval from the preceding child? | __________ Month |  |
| **Common childhood illnesses in the last 2 weeks** | | | |
| **C8** | Cough | 1. Yes 0. No |  |
| **C9** | Diarrhea (Three or more loose or watery stool within 24hour) | 1. Yes 0. No |  |
| **C10** | Fever | 1. Yes 0. No |  |
| **C11** | Others | Specify_______________________ |  |
| **Child Feeding Practice** | | | |
| **C12** | How soon after giving birth did the baby first receive breast-milk? | 1. Immediately 2. 30 minutes later 3. One hour later 4. Others______________ |  |
| **C13** | Did the baby receive the first milk (colostrum)? | 1. Yes 0. No |  |
| **C14** | In the first three days after delivery, did you gave pre-lacteal feeding to your child? | 1. Yes 0. No |  |
| **C15** | During the first 6 months of life did your baby receive any liquids or solid, except drugs, vitamins prescribed by health professionals | 1. Yes 0. No |  |
| **C16** | Are you breastfeeding now | 1. Yes 0. No |  |
| **C17** | If no when did you stop breastfeeding  (Child age in a month) | _________________________ |  |
| **C18** | Did the child start complementary feeding? | 1. Yes 0. No | If No to C21 |
| **C19** | At what age did you start giving other liquids or food to your baby? | __________________________ |  |
| **C20** | What do you mainly use to feed your child? | 1 .Bottle  2. Cup  3. Spoon  4. Other (specify) __________ |  |

**Child Dietary Diversity Score:**

| Now I would like to ask you about the types of foods that the child included in the study ate yesterday during the day and at night. Say YES if the child ate the food in the question. Say NO if she/he does not eat the food in the question. | | | |
| --- | --- | --- | --- |
|  | **Food groups** | **List of foods** | **1. Yes 0. No** |
| **C21** | Grains, roots, and tubers | Porridge, bread, rice, noodles, or other foods made from grains  White potatoes, white yams, cassava, or any other foods made from roots |  |
| **C22** | Legumes and nuts | Any foods made from beans, peas, lentils, nuts, or seeds |  |
| **C23** | Dairy products | Infant formula, Milk, such as tinned, powdered or fresh animal milk, Yogurt or drinking yogurt Cheese or other dairy products |  |
| **C24** | Flesh foods | Liver, kidney, heart or other organ meats, Any meat, such as beef, lamb, goat, chicken, Fresh or dried fish |  |
| **C25** | Egg | Eggs |  |
| **C26** | Vitamin A-rich fruits and vegetables | Pumpkin, carrots, squash, or sweet potatoes that are yellow or orange inside  Any dark green vegetables, Ripe mangoes (fresh or dried [not green]), ripe papayas (fresh or dried)  Foods made with red palm oil, red palm nut, or red palm nut pulp sauce |  |
| **C27** | Other fruits and vegetables | Any other fruits or vegetables (tomato, kale, salad, sugar beet, avocado, banana, orange, lemon…) |  |

**Section D: Maternal Related Factors**

| **Name** | **Variable** | **Response** | | **Skip** |
| --- | --- | --- | --- | --- |
| **D1** | Did you attend ANC service for the selected child? | 1. Yes 0. No | | If No to D3 |
| **D2** | How many times did you attend the ANC service | ______________ | |  |
| **D3** | When do you usually wash your hands? (More than one answer is possible) | 1. After latrine use  2. Before preparing food  3. Before serving food  4. Other (specify)__________ | |  |
| **D4** | How do you wash your hand? | 1. Always with water and soap 2. Always with only water 3. Always with water and sometimes with water & soap 4. Sometimes eat without washing my hands | |  |
| **Women Autonomy questions: Ask the mother of the child** | | | | |
| **Freedom of Movement** | |  | |  |
| **D5** | Do you have to ask your husband or a senior family member for permission to go anyplace outside your house or compound? | 1= Yes 0= No | |  |
| **D6** | Do you have to ask your husband or a senior family member for permission to go to the local health center? | 1= Yes 0= No | |  |
| **D7** | Do you have to ask your husband or a senior family member for permission to go to the local market? | 1= Yes 0= No | |  |
| **Decision-Making Regarding Children: Please tell me who in your family decides the following** | | | | |
| **D8** | What to do when a child falls sick? | 1= Only my husband  2= Both jointly  3= I myself  4= Others, ( specify)________ | |  |
| **D9** | How much schooling to give to your children? | 1= Only my husband  2= Both jointly  3= I myself  4= Others, ( specify)________ | |  |
| **D10** | To whom to marry your children (probe the mother for current (if exist) and or expectation in future)? | 1= Only my husband  2= Both jointly  3= I myself  4= Others, ( specify)________ | |  |
| **Household Tasks and Decisions (final say)** | | | | |
| **D11** | What food to buy for family meals | 1= Only my husband  2= Both jointly  3= I myself  4= Others, ( specify)________ | |  |
| **D12** | Whether to purchase major goods for the household such as oxen, land, and house | 1= Only my husband  2= Both jointly  3= I myself  4= Others, ( specify)________ | |  |
| **Autonomy regarding Family planning service utilization** | | | | |
| **D13** | Can you decide the number of children you need to have alone | 1= Yes 0= No | |  |
| **Maternal knowledge of child feeding practice** | | |  |  |
| **D14** | A neonate should start breastfeeding within 1 hour of birth | **0**= No **1**= Yes **2**= I don’t know | |  |
| **D15** | An infant should exclusively breastfeed for the first 6 months | **0**= No **1**= Yes **2**= I don’t know | |  |
| **D16** | An infant should breastfeed at least 8 times/day (on-demand but not <8 times) | **0**= No **1**= Yes **2**= I don’t know | |  |
| **D17** | An infant should finish one breast milk before switching to other breasts | **0**= No **1**= Yes **2**= I don’t know | |  |
| **D18** | An infant should continue breastfeeding until 2 or more years | **0**= No **1**= Yes **2**= I don’t know | |  |
| **D19** | An infant should start complementary foods at 6 months | **0**= No **1**= Yes **2**= I don’t know | |  |
| **D20** | A breastfeed 6–8 months infant should take complementary food 2–3 times/day | **0**= No **1**= Yes **2**= I don’t know | |  |
| **D21** | A breastfeed 9–12 months infant should take complementary food 4 times/day | **0**= No **1**= Yes **2**= I don’t know | |  |
| **D22** | A 6–23-month child needs 4 or more food staffs | **0**= No **1**= Yes **2**= I don’t know | |  |
| **D23** | Non breastfeed infant needs an extra meal | **0**= No **1**= Yes **2**= I don’t know | |  |
| **D24** | An infant needs iron-rich foods | **0**= No **1**= Yes **2**= I don’t know | |  |
| **D25** | Sick children need an extra meal | **0**= No **1**= Yes **2**= I don’t know | |  |
| **D26** | Source of information for the above questions | 1. Health professionals 2. Mass media 3. Relatives | |  |

**Mothers Dietary Diversity Score:**

| Yesterday during the day or at night, what did you eat, whether you ate it at home or anywhere else? Morning, mid-morning, noon, afternoon, evening, late evening | | | |
| --- | --- | --- | --- |
|  | **Food groups** | **List of foods** | **1. Yes**  **0. No** |
| **D27** | Grains, white roots, and tubers | Porridge, bread, rice, noodles, or other foods made from grains  White potatoes, white yams, cassava, or any other foods made from roots |  |
| **D28** | Pulses (beans, peas, and lentils) | Mature beans or peas (fresh or dried seed), lentils, or bean/pea products |  |
| **D29** | Nuts and seeds | Any tree nut, groundnut/peanut or certain seeds, or nut/seed “butter” or pastes |  |
| **D30** | Dairy products | Milk, cheese, yogurt, or other milk products but not including butter, |  |
| **D31** | Meat, poultry, and fish | Liver, kidney, heart, or other organ meats. Any meat, such as beef, lamb, goat, chicken, Fresh or dried fish  Liver, kidney, heart, or other organ meats, including wild game, Beef, lamb, goat, wild game meat, chicken, or other bird  Fresh or dried fish |  |
| **D32** | Egg | Eggs from poultry or any other bird |  |
| **D33** | Dark green leafy vegetables | List examples of any medium-to-dark green leafy vegetables, including wild/foraged leaves |  |
| **D34** | Another vitamin A-rich fruits & vegetables | Pumpkin, carrots, squash, or sweet potatoes that are yellow or orange inside Ripe mango, ripe papaya |  |
| **D35** | Other vegetables | Vegetables ( tomato, kale, salad, sugar beet,) |  |
| **D36** | Other fruits | Fruits (avocado, banana, orange, lemon) |  |

**Section F: Household Food Insecurity Access Scale and Related Question**

|  | **Question** | **Response** |
| --- | --- | --- |
| **F 1** | In the past four weeks, did you worry that your household would not have enough food | 1=Yes  0=No |
| **F _1.1_** | If yes, how often did this happen? | 1 = Rarely (1-2times)  2 = Sometimes (3 to 10 times)  3 = Often (more than 10 times) |
| **F 2** | In the past four weeks, were you or any household member not able to eat the kinds of foods you preferred because of a lack of resources? | 1=Yes  0=No |
| **F _2.1_** | If yes, how often did this happen? | 1 = Rarely (1-2times)  2 = Sometimes (3 to 10 times)  3 = Often (more than 10 times) |
| **F 3** | In the past four weeks, did you or any household member have to eat a limited variety of foods due to a lack of resources? | 1=Yes  0=No |
| **F _3.1_** | If yes, how often did this happen? | 1 = Rarely (1-2times)  2 = Sometimes (3 to 10 times)  3 = Often (more than 10 times) |
| **F 4** | In the past four weeks, did you or any household member have to eat some foods that you really did not want to eat because of a lack of resources to obtain other types of food? | 1=Yes  0=No |
| **F _4.1_** | If yes, how often did this happen? | 1 = Rarely (1-2times)  2 = Sometimes (3 to 10 times)  3 = Often (more than 10 times) |
| **F 5** | In the past four weeks, did you or any household member have to eat a smaller meal than you felt you needed because there was not enough food? | 1=Yes  0=No |
| **F _5.1_** | If yes, how often did this happen? | 1 = Rarely (1-2times)  2 = Sometimes (3 to 10 times)  3 = Often (more than 10 times) |
| **F 6** | In the past four weeks, did you or any household member have to eat fewer meals in a day because there was not enough food? | 1=Yes  0=No |
| **F _6.1_** | If yes, how often did this happen? | 1 = Rarely (1-2times)  2 = Sometimes (3 to 10 times)  3 = Often (more than 10 times) |
| **F 7** | In the past four weeks, was there ever no food to eat of any kind in your household because of a lack of resources to get food? | 1=yes  0=no |
| **F _7.1_** | If yes, how often did this happen? | 1 = Rarely (1-2times)  2 = Sometimes (3 to 10 times)  3 = Often (more than 10 times) |
| **F 8** | In the past four weeks, did you or any household member go to sleep at night hungry because there was not enough food? | 1=Yes  0=No |
| **F _8.1_** | If yes, how often did this happen? | 1 = Rarely (1-2times)  2 = Sometimes (3 to 10 times)  3 = Often (more than 10 times) |
| **F 9** | In the past four weeks, did you or any household member go a whole day and night without eating anything because there was not enough food? | 1=Yes  0=No |
| **F _9.1_** | If yes, how often did this happen? | 1 = Rarely (1-2times)  2 = Sometimes (3 to 10 times)  3 = Often (more than 10 times) |
| **Household Food Insecurity Related Question** | | |
| **F 10** | Do you use productive safety-net service | 1. Yes 0. No |

**Section G: Anthropometric Measurement**

| **Child**  **Anthropometry** | **Weight (KG)** **=** ***⎥______________________⎥***  **Height/Length (CM)= *⎥ _________________⎥*** | ***(Length*** *for children under 2 years of age****)***  ***(Height*** *for children 2 years and above****)*** |
| --- | --- | --- |
| **Check for pitting edema of both feet** | **Yes_______ No____________** |  |

***Thank you for your participation!!***
